# Report of Four Cases of Laparotomy, Two Cases of Intra-Ligamentous Cyst, One Dermoid Cyst and One Removal of Appendages, Two of the Cases Requiring Hysterectomy

**Published:** 1890-05

**Authors:** X. O. Werder

**Affiliations:** Pittsburgh


### REPORT OF FOUR CASES OF LAPAROTOMY; TWO CASES OF INTRA-LIGAMENTOUS CYST, ONE DERMOID CYST AND ONE REMOVAL OF APPENDAGES, TWO OF THE CASES REQUIRING HYSTERECTOMY.*

BY X. O. WERDER, M. D., PITTSBURGH.

I.—Tait's operation. Patient thirty-eight years of age, has had five children, the youngest of which is nine years old. Since the birth of the last child she has never been well, suffering with constant pelvic symptoms and reflex neuroses, especially severe pains about the left side of the chest; had been treating her for more than two months with hypodermic injections of morphine, gr. ¾ often being required to relieve her. Vaginal examination revealed an adherent retroflexed uterus, with great tenderness of the uterine adnexa.

Laparotomy was performed December 7th, 1889. The retroflexed uterus, which was held in its abnormal position by small fibrinous bands, was replaced, and the ovaries and tubes on both sides were removed. I had intended to perform a hysterorraphy at the same time, in order to prevent displacement of the uterus, but after removing the appendages close to the uterus, taking in the slack in the broad ligaments, I found the uterus in perfectly normal position, so that I did not think it necessary to do anything further. The patient made an uninterrupted recovery, the temperature never going beyond 99.4°. The veins in the broad ligaments were varicosed, containing a number of phleboliths. The tubes were somewhat thickened; the fimbriæ destroyed. The right tube was adherent to the ovary, but the adhesions were separated during the operation; both tubes and parts of the broad ligaments were studded with small cysts. The ovaries were cyrrhotic, the left one very small and hard, the right one consisting chiefly of a number of cysts, very little of the ovarian

*Read before the Allegheny County Medical Society, March 18, 1890.

stroma remaining intact. While the pelvic symptoms were completely relieved, the reflex neuroses were improved but not cured up to the present time.

II.—Mrs. D., 42 years old, no children; was suffering with a right ovarian cyst, which had been growing for about four years. Had also an adherent retroflexed uterus very much enlarged. For some years she had been subject to severe menorrhagia, which during the last four months had become so severe that she was obliged to remain in bed almost half her time. These hemorrhages had weakened her down very much; she was very anæmic and had the appearance of a woman of about sixty. Operation was performed January 9th, 1890, Dr. J. J. Buchanan assisting. I found an intra-ligamentous ovarian cyst, the capsule completely enveloping the cyst except on its anterior aspect; the largest part of the tumor was very low down in the pelvis. The tumor was shelled out from its capsule, which, however, proved an exceedingly difficult task, as the walls of both cyst and capsule were very thin, causing them to tear through quite frequently; they were also very firmly adherent, rendering the operation very difficult and tedious. Some of the adhesions were very vascular; the hemorrhage during the operation was truly frightful; once or twice the blood welled up from the pelvis in such quantities that I feared I had torn the iliac veins. Most of the operation had to be done by the fingers, unaided by sight, as the tumor was so deep that nothing could be seen. During the dissection my fingers picked up the right ureter, which was firmly adherent to the tumor, but which I succeeded in separating without injury to the ureter. On two occasions the patient was pulseless during the operation, but was revived by hypodermics of whiskey, of which several dozen were given. The operation, from the time she was placed on the table until she was removed to bed, lasted almost one and a half hours. After the tumor had been removed I washed her out freely with hot water, which returned perfectly clear; this was repeated after placing the stitches. A pretty large quantity of water was left in the abdominal cavity to counteract the great loss of blood; a drainage tube was inserted. The patient's pulse had considerably improved upon the abdominal flushings, but shortly after it commenced to get weaker and weaker in spite of stimulation by mouth and hypodermically, and she died within ten hours after operation, from shock, never having rallied therefrom. About two hours after operation she had a violent fit of vomiting, which expelled a considerable quantity of bloody fluid from the drainage tube, saturating even the outer dressings, but when the nozzle of a syringe was passed down into the abdominal cavity and operated, there was only a small quantity of dark blood withdrawn, showing that there had been no new bleeding, and that the fluid expelled from the tube was simply the water left in the abdominal cavity stained with blood.

III.—Large dermoid cyst. Hysterectomy. Mrs. G., 52 years of age, is the mother of a large family. Two years ago menopause became established. About 10 months ago she noticed the appearance of a tumor which increased very rapidly in size ; for about four weeks before operation she was hardly able to leave the bed. She had several attacks of profuse uterine hemorrhage during that time, it being the first show for two years.

Laparotomy was performed January 22, 1890. Found a large dermoid cyst of the size of a uterus at six months' pregnancy, with universal adhesions, especially to mesentery, omentum, intestines, sigmoid flexure, and pelvic walls. Upon emptying the cyst and separating the adhesions, the tumor was brought up into the abdominal wound, when it was found that a large portion of the base of it was a solid mass which was attached to the right side of the fundus uteri. This having been detached, it was found that the uterus itself was disintegrated to such an extent that, in removing the diseased mass, I removed a part of the uterine wall, leaving a large ulcerated cavity with ragged edges and filled with a soft, friable and cheesy mass which extended almost to the endometrium. There was no bleeding from this surface whatever. A hysterectomy, therefore, became necessary. An elastic ligature was passed around the cervix, the body removed, and the pedicle brought into the lower angle of the abdominal wound. There was still some free bleeding deep down in the pelvis, apparently coming from some vessels in the sacro-uterine ligaments which had been torn in bringing up the uterus. It was exceedingly difficult to reach the source of the hemorrhage, but at last I succeeded in grasping the bleeding points with two large Spencer Well's pressure forceps which I left attached, the handles being left outside of the abdomen. These were removed on the second and third days respectively.

The contents of the cyst were a thick creamy fluid looking exactly like pus, a large bunch of hair, and one little piece of bone attached to the internal cyst-wall. The abdomen was well flushed with hot water. No drainage tube was inserted, as a glass drainage tube in such close proximity to the two large clamp-forceps in the abdominal cavity seemed rather risky. The abdominal wound was closed in the usual way.

This patient, from which everybody present at the tru y frightful operation, including herself, gave a fatal prognosis, rallied well from the shock and made a good recovery, her convalescence at no time being complicated by any untoward symptoms. The portion of the tumor attached to the uterus which has become softened and broken down, involving the uterine structure with its endometrium, the uterus itself being greatly enlarged, had all the appearances of malignant disease, but according to the microscopical examination of Dr. Matson, it fortunately proved to be a fibroma which, I suppose, had undergone a process of disintegration. This condition of the uterus sufficiently explains the hemorrhages which the patient had been subject to during the last few weeks preceding the operation.

IV.—Intra-ligamentous cyst. Ovariotomy and hysterectomy. Mrs. G., referred to me for operation by Dr. J. M. Stevenson, had a large ovarian tumor, which had been growing for quite a time, but which had increased more rapidly during the last few months, so that it had attained the size of a full-grown pregnancy. Though 67 years of age and mother of a grown-up family, her physical condition was good, and she was regarded by her physician, Dr. Stevenson and myself, a fair subject for operation.

The operation was performed January 25, 1890. No adhesions were encountered; the tumor was emptied and drawn out of the abdominal cavity, but when the pedicle was reached it was found extremely large and thick, and on closer examination a part of it proved to be the uterus. It was an intra-ligamentous cyst so closely attached with its lower portion to the uterus that it was thought preferable to remove the uterus with the cyst, than to attempt any enucleation, as this certainly would have been exceedingly difficult, and could not have been accomplished without a great deal of hemorrhage, as the parts were exceedingly vascular, and the veins very much dilated. It was therefore thought less risky, considering the age of the patient, to remove the uterus with the pedicle, than to expose her to the danger of an exhausting hemorrhage. This was done without any bleeding whatever, so that the usual toilet of the peritoneum, or flushing of the abdominal cavity, was omitted. The uterine stump was treated as in the first case, with an elastic ligature, and brought out to the lower angle of the wound, uniting peritoneum below ligature, so as to shut out the pedicle from the abdominal cavity. The patient made an elegant and uninterrupted recovery. There was one peculiarity about this uterus. The tumor being so large that it was completely drawn out of the pelvis, necessarily drew the firmly-attached uterus with it, which caused such a stretching of that organ that the lower portion of it felt like a long, hard cord included in the pedicle of the tumor. This had the effect also of stretching and elongating the vagina, the upper part of it being very much contracted and funnel-shaped, the uterus being entirely out of reach.

Neither of these two cases of hysterectomy, even the one who had the large clamp forceps in the abdominal cavity for three days, ever required a single dose of opium or morphine to relieve pain; both seemed to be perfectly comfortable and contented.

